# 

**Published:** 1874-05

**Authors:** 


### ASSOCIATIONS.

We take pleasure in calling the attention of the profession, and especially those of the Eastern States, to the following announcement. It will certainly be a good meeting, and all who are within reach, and have the opportunity should attend. We have met almost all of the gentlemen of these societies, and know personally that a meeting with them portends good.

### CONN. VALLEY DENTAL ASSOCIATION.

The Massachusetts Dental Society has accepted the invitation of the Connecticut Valley Dental Association, and will meet in convention, at Ingleside Hotel, Tuesday and Wednesday, June 9th and 10th, 1874.

The following Committees have been appointed, and discussions will follow each report in order :

Mechanical Dentistry.—C. S. Hurlbut, Springfield, Mass.
Dental Appliances.—J. F. Adams, Worcester, Mass.
Operative Dentistry.—Geo. L. Parmelee, Hartford, Conn.
Dental Medicines.—H. F. Bishop, Worcester, Mass.
Treatment of exposed Pulps.—F. Searle, Springfield, Mass.
Dental Pathology.—C. A. Brackett, Newport, R. I.
Dental Education.—E. G. Leach, Boston, Mass.
Microscopy.—S. B. Bartholomew, Springfield, Mass.
Dental Literature.—T. B. Hitchcock, Boston, Mass.

Gentlemen having Volunteer Essays, will please report to H. F. Bishop, Chairman of Executive Committee.

L. C. Taylor, Secretary.

### INDIANA STATE DENTAL SOCIETY.

The annual meeting of the Indiana State Dental Society will be held in Indianapolis, on Tuesday the 30th of June, prox.

We make this statement thus early, that every dentist in the state, and we trust many outside of the State, will make their arrangements to be present and participate in the proceedings. All who attend this society may entertain a certainty of a good time socially, and an interesting and profitable time professionally. So come one and all. We shall give the regular programme in the next No.

### IOWA STATE DENTAL SOCIETY.

The twelfth annual meeting of this Society will be held at Dubuque, May 19th, 1874:

W. P. Dickinson, Dubuque; M. D. Globe, Dubuque; H. W. Ray, Bellevue; Executive Committee.

The Iowa State Society is a very effiicient society, and merits the hearty co-operation of the profession of that state; and every member of the profession in the state, who is susceptible of instruction, or is able to communicate information to others, should attend. This does not apply to nor is it intended for those who do not possess either of the above requisites. They may stay at home, as they usually do. Let this meeting be a rousing outpouring of the profession in all that country.

### PROCEEDINGS.

We intended to publish in this number of the REGISTER the proceedings of the Mississippi Valley Dental Association, but we did not receive them till about the going to press, and we could not hold back for them. We hope to publish them in the June No.

---

MARRIED: April 28th, 1874, by Rev. Geo. C. Heckman, D. D., assisted by Rev. J. F. Hutchison, at Madison Ind.; Wm. A. Graham, D. D. S., and Mary J. Hanley—both of that city.

The happy pair have our warmest wishes for the future. The Dr. evidently entered upon this matter after mature deliberation, and he will no doubt be all the happier for it.

---

### ILLINOIS STATE DENTAL SOCIETY.

We give below the programme of the tenth annual meeting of this society which will be held on the twelfth and fifteenth inst inclusive, at Jacksonville, begining at 10 o'clock A. M. This is one of the most active and best state societies in the country. Its published transactions gives evidence of this; but then any one will readily understand that such publications give but a very feeble idea of the life and spirit of such a meeting. The social element and stimulating influence that exists and is operating upon such occasions, can not be transmitted through publications.

The Illinois Society is one of the largest, if not the largest in the country, and embraces many of the best men of the profession in its ranks.

A general invitation is extended to the profession, and we will suggest that no one who can possibly reach it can afford to stay away. But to the programme.

### ILLINOIS STATE DENTAL SOCIETY.

*Order of Business.*

FIRST DAY, MORNING SESSION.

1. Meeting of Executive Committe, Treasurer and Secretary, for payment of dues and other preliminary business.
2. Organization, Roll-call and reading of the Records.
3. Applications for Membership.
4. Reports of Committees.
5. Reports of Officers.
6. Election of Members.
7. Miscellaneous Business.
8. Adjournment to 2 o'clock.

FIRST DAY, AFTERNOON SESSION.

1. Reading Minutes of Morning Session.
2. Annual Address by the President.
3. Discussion of topics selected, Essays upon which will be read by the gentlemen whose names are thereunto annexed.

FIRST DAY, EVENING SESSION.

Essays and Discussion.

SECOND DAY, MORNING SESSION.

Clinics—Conducted by H. H. Townsend, Pontiac; E. Honsinger Chicago; Edgar Fark, St. Louis; G. S. Miles. Jerseyville, Warren DeCrow, Quincy.

SECOND DAY, AFTERNOON SESSION.

1. Presentation and Discussion of Scientific Experiments, or other subjects of special interest not coming under the head of the subjects announced.
2. Continuation of Essays and Discussions.

SECOND DAY, EVENING SESSION.

Essays and Discussion.

THIRD DAY, MORNING SESSION.

Clinics Conducted by G. O. Howard, Galena; J. M. Hurtt, Peoria; Prof. W. H. Eames, St. Louis; W. W. Ormsbee, Geneva; M. S. Dean, Chicago.

THIRD DAY AFTERNOON SESSION.

Essays and Discussion.

THIRD DAY, EVENING SESSION.

Essays and Discussion.

FOURTH DAY, MORNING SESSION.

Essays and Discussion.

FOURTH DAY, AFTERNOON SESSION.

1. Essays and Discussion until 4 o'clock.
2. Selection of place of next meeting.
3. Election of Officers.
4. Report of Committees and Miscellaneous Business.
5. Adjournment.

*Subjects for Discussion.*

1. Dental Students.

C. A. Kitchen, Galva.

2. Why so many failures in Dental operations.

C. R. Dwight, Danville.

3. Thoroughness and honesty in Dental operations.

H. H. Townsend, Pontiac.

4. Ætiology of Dental Caries.

A. W. Harlan, Chicago.

5. Treatment of Exposed Pulps.

J. N. Crouse, Chicago.

6. What changes should be made in the shapes of teeth in treating decay, and how to make them.

Edmund Noyes, Chicago.

7. Sixth Year Molars.

G. S. Miles, Jerseyville.

8. Filling Bicuspids.

W. S. Ritchey, Chicago.

9. The duty of Dentists in teaching their patrons in the general care of their teeth.

J. F. Marriner, Ottawa.

The Executive Committee can be found at the Dunlap House on the evening preceding the meeting, and until 10 o'clock Tuesday morning. G. V. Black,

*Chairman Executive Committee.*

### CONTENTS.

### COMMUNICATIONS.

Abnormal Mucous Membrane...... ................ 185
Revelations of the Microscope...................... 193
Hernia of the Dental Pulp......................... 198
Address to the graduates of the Ohio College of Dental Surgery............................................ 203
Reply on behalf of the class of 1874.................. 211

### CORRESPONDENCE.

Letter from China........ ......................... 214
Revival in Dental Journalism.................... ..... 217

### EDITORIAL.

To our Contributors............ ................... 219

To the Profession............................ ..... 220

Associations...................................... 223

History of Dentistry .............................. 225

Illinois State Dental Society......................... 226

### THE DENTAL REGISTER,

### OF THE WEST.

### A Monthly Magazine, Devoted Entirely to the Interest of the DENTAL PROFESSION.

**Terms Reduced to $2.50 per Year. Single Nos. 25 Cents.**

**Edited by PROF. J. TAFT,**

**And Published by SPENCER & MOORE, of the Ohio Dental Depot**

The volume commences in January and closes in December.

The Register being issued monthly is always fresh, therefore indispensable to the practitioner who desires to keep posted up with the new inventions and improvements which are daily developed. Neither expense nor effort will be spared to give value and variety to its contents. Articles will be illustrated with well executed engravings whenever they will add to the interest or assist in explanation.

Its extensive circulation and frequency of issue makes it one of the BEST DENTAL ADVERTISING MEDIUMS in the United States.

All subscriptions must begin with January or July.

**RATES OF ADVERTISING:**

| | 1 mo. | 3 mo. | 6 mo. | 1 year. | | 1 year. | |
|---|---|---|---|---|---|---|---|
| 1 page ......... | $12 00 | $25 00 | $40 00 | $75 00 | Inserted pages must be plain paper & black ink. | 1st page. | $50 00 |
| ½ page ......... | 8 00 | 15 00 | 25 00 | 40 00 | | 2d page. | 37 50 |
| ¼ page ......... | 5 00 | 10 00 | 15 00 | 25 00 | | 3d page. | 33 33 |
| | | | | | | 4th page. | 25 00 |

Local or special notices, five lines or less, will be inserted for $3 each insertion; over five lines, 50 cents per line.

All advertising bills payable in advance, or at furthest, after the issue of the first number containing the advertisement. All transient advertisements to be paid for in advance.

☞CONTRIBUTIONS SOLICITED.

Exclusively Editorial Communications should be addressed to

**DR. J. TAFT, EDITOR.**

The latest number of the Register may be ordered with or without other goods at any time, and all letters should be addressed to

**SPENCER & MOORE. PUBLISHERS,**

Cincinnati, Ohio.

